# Prevalence and Risk Factors of Oral Cancer Among Dental Students From Two Private Colleges in Pakistan: A Comparative Study

**DOI:** 10.7759/cureus.71332

**Published:** 2024-10-12

**Authors:** Atiq Ur Rehman, Hafiz Nasir Mahmood, Aneela Bashir, Mariyah Javed, Muhammad Anwaar Alam, Ali Anwaar

**Affiliations:** 1 Department of Prosthodontics, Institute of Dentistry, Combined Military Hospital (CMH) Lahore Medical College, Lahore, PAK; 2 Department of Oral and Maxillofacial Surgery, Institute of Dentistry, Combined Military Hospital (CMH) Lahore Medical College, Lahore, PAK; 3 Department of Oral Pathology, Abbottabad International Medical Institute, Abbottabad, PAK; 4 Department of Oral Pathology, Rahbar College of Dentistry, Lahore, PAK; 5 Department of Oral Biology, Azra Naheed Dental College, The Superior University, Lahore, PAK; 6 Department of Community and Preventive Dentistry, Institute of Dentistry, Combined Military Hospital (CMH) Lahore Medical College, Lahore, PAK

**Keywords:** betel quid chewing, dietary habits, oral cancer, pakistan, student health, tobacco use

## Abstract

Background: Globally, oral cancer is still a major public health concern, with different lifestyle and socioeconomic variables influencing its occurrence.

Objective: This study compared the prevalence and risk factors of oral cancer among students from private colleges in Pakistan, identifying patterns and variations in risk profiles within this group.

Methodology: This six-month cross-sectional research was carried out at Azra Naheed Dental College and Rahbar College of Dentistry, two private dental institutions in Lahore, Pakistan. Four hundred and thirty students between the ages of 18 and 30 who were split evenly between the two schools made up the sample. A well-structured questionnaire including lifestyle variables, oral cancer awareness, and demographic information was used to gather data. Dental practitioners with training conducted clinical oral exams to look for any indications of oral lesions. The demographic variables were summed up using descriptive statistics, and the relationships between risk factors and the prevalence of oral cancer were assessed using multivariate logistic regression analysis. A p-value of less than 0.05 was used to define statistical significance.

Results: Among the 430 students, 215 were from Azra Naheed Dental College and 215 were from Rahbar College of Dentistry; 12 students (5.58%) at Azra Naheed and 19 students (8.84%) at Rahbar College were found to have oral lesions. Regarding lifestyle factors, 69 students (32.22%) at Azra Naheed and 62 students (28.89%) at Rahbar College reported current tobacco use. Regular betel quid chewing was observed in 93 students (43.33%) at Azra Naheed compared to 77 students (35.56%) at Rahbar College. Dietary habits showed that 148 students (68.89%) at Azra Naheed and 138 students (64.44%) at Rahbar College consumed a diet high in processed foods. The multivariate logistic regression analysis identified significant risk factors for oral cancer, including current tobacco use (odds ratio (OR) = 2.15, p = 0.001), regular betel quid chewing (OR = 2.87, p < 0.001), and a diet high in processed foods (OR = 1.62, p = 0.021).

Conclusion: The research identifies important risk factors for oral cancer among dentistry college students, pointing to the need for focused preventative and educational initiatives to address lifestyle-related concerns.

## Introduction

The global morbidity and death rate from oral cancer makes it a serious public health concern [1, 2]. Concern about this cancer's increasing incidence in Pakistan is growing, especially in light of the nation's distinct socioeconomic and cultural characteristics that may affect the disease's occurrence as well as its treatment [3, 4].

Tobacco usage, chewing betel quid, and dietary practices are some of the factors that influence the prevalence of oral cancer in Pakistan and have all been linked to higher risk [5]. Notwithstanding these established correlations, a dearth of thorough, comparative studies persists on the potential impact of disparate educational settings, including private universities, on the incidence and awareness of oral cancer in young people [6].

The research on oral cancer frequency and risk variables is made more interesting by the distinctive setting offered by Pakistan's private colleges, which serve a wide range of socioeconomic backgrounds [7, 8]. These educational establishments often cater to students from many origins and locations, providing a cross-sectional perspective of the populace that may illuminate regional and demographic differences in the incidence of oral cancer [9]. Moreover, students attending private colleges may have a very different lifestyle and level of health consciousness than students attending public universities, which might have an impact on their risk factors and preventative measures [10, 11].

Gaining knowledge on the incidence of oral cancer and the risk factors linked to it in these educational environments may be very beneficial in determining the disease's spread and the efficacy of existing preventative measures. This information is essential for creating population-specific preventative initiatives and health education campaigns.

This research examined the frequency and risk factors for oral cancer among Pakistani students attending private colleges, finding trends and differences in their risk profiles.

## Materials and methods

Study design and setting

This cross-sectional research examined the risk factors and prevalence of oral cancer, specifically focusing on squamous cell carcinoma (SCC), among Pakistani students attending private institutions. The study was conducted at two locations over six months, from January 2024 to June 2024: Rahbar College of Dentistry and Azra Naheed Dental College in Lahore, Pakistan. These universities were chosen to provide a diverse student body for analyzing differences in the incidence of oral cancer and related risk factors.

Inclusion and exclusion criteria

The research included students aged between 18 and 30 who were enrolled in Rahbar College of Dentistry or Azra Naheed Dental College during the study period. Informed consent was obtained from all participants. Students with a history of oral cancer or serious oral disorders that might skew the research findings were excluded from participation.

Sample size

There were 506 students in the study at first. Out of them, 76 students were disqualified because of the following reasons: 28 did not provide permission, 31 were absent while the data were being collected, and 17 had a history of oral illnesses. As a result, 430 students made up the total sample size. In order to guarantee strong outcomes, the final sample was split equally between Rahbar College of Dentistry and Azra Naheed Dental College, with 215 students from each school. While taking into consideration differences in the demographics and lifestyles of students, this distribution offered a fair picture of the prevalence and risk factors of oral cancer.

Data collection

Data were collected through clinical oral examinations and a standardized questionnaire. The clinical exams were conducted by trained dental practitioners who assessed participants for signs of oral lesions, pre-cancerous changes, and other pathological findings in the oral cavity. In cases where lesions were detected, further diagnostic procedures, such as biopsy, were recommended to confirm the diagnosis histologically. Participants provided informed consent for their data to be used in the research.

The standardized questionnaire included sections designed to quantify dietary habits and lifestyle factors. Respondents reported their consumption frequency of specific food categories, including processed foods, fruits, and vegetables, using the options of daily, weekly, occasionally, or never. Processed foods encompassed items such as chips, sugary snacks, and fast foods. For fruits and vegetables, participants specified their intake of various types, providing insight into their dietary patterns in relation to oral health and cancer risk. Additionally, the questionnaire assessed lifestyle variables, such as chewing betel quid, smoking, and tobacco use, along with participants' awareness of oral cancer.

Statistical analysis

IBM SPSS Statistics software, version 26 (IBM Corp., Armonk, NY) was used to examine the data. Risk variables, prevalence rates, and demographic features were compiled using descriptive statistics. The study used inferential statistics, specifically multivariate logistic regression analysis, to ascertain noteworthy correlations between risk variables and the incidence of oral cancer. A significance threshold of p < 0.05 was used.

Ethical approval

Ethical approval was obtained from the Institutional Review Board (IRB) of both Azra Naheed Dental College and Rahbar College of Dentistry (approval number: ANDC/RAC/2023/34). Informed consent was secured from all participants, and confidentiality of their data was maintained throughout the study. The research adhered to ethical guidelines and standards for human research.

## Results

Of the 215 students enrolled at Azra Naheed Dental College, 92 (42.79%) were between the ages of 18 and 22, 76 (35.35%) were between the ages of 23 and 26, and 47 (21.86%) were between the ages of 27 and 30 (Table 1). There were 91 female students (42.33%) and 124 male students (57.67%). Of the students, 79 were in their first year (36.75%), 69 were in their second year (32.19%), and 67 were in their third year (31.16%). Socioeconomically speaking, 110 students (51.16%) came from a moderate socioeconomic background, whereas 69 students (32.09%) came from a low socioeconomic background. By contrast, Rahbar College of Dentistry (n = 215) had 51 (23.72%) students aged between 27 and 30 years, 57 (26.51%) students aged between 23 and 26 years, and 107 (49.77%) students aged between 18 and 22 years. There were 113 male students (52.56%) and 102 female students (47.44%) in this class; 81 students (37.67%) were in their second year, 72 (33.49%) were in their third year, and 62 (28.84%) were in their first. Socioeconomically speaking, 103 students (47.91%) came from a moderate socioeconomic background, whereas 81 students (37.68%) came from a low socioeconomic background.

**Table 1:** Demographic characteristics of the study participants

Characteristic		Azra Naheed Dental College (n = 215)	Rahbar College of Dentistry (n = 215)
Age (years)	18-22	92 (42.79%)	107 (49.77%)
	23-26	76 (35.35%)	57 (26.51%)
	27-30	47 (21.86%)	51 (23.72%)
Gender	Male	124 (57.67%)	113 (52.56%)
	Female	91 (42.33%)	102 (47.44%)
Year of study	First year	79 (36.75%)	62 (28.84%)
	Second year	69 (32.09%)	81 (37.67%)
	Third year	67 (31.16%)	72 (33.49%)
Socioeconomic status	Low	69 (32.09%)	81 (37.68%)
	Middle	110 (51.16%)	103 (47.91%)
	High	36 (16.75%)	31 (14.41%)

Table 2 shows that among the students enrolled at Azra Naheed Dental College (n = 215), 69 (32.22%) were using tobacco, 31 (14.44%) had used it in the past, and 115 (53.33%) had never used it. Regarding the habit of chewing betel quid, 93 students (43.33%) chewed daily, 43 students (20.00%) chewed on occasion, and 79 students (36.67%) never chewed it. In terms of eating habits, 148 students (68.89%) had a diet heavy in processed foods, whereas 67 students (31.11%) had a diet high in fruits and vegetables. Among the students at Rahbar College of Dentistry (n = 215), 134 (62.22%) had never used tobacco, 19 (8.89%) were past users, and 62 (28.89%) were current users. Chewing betel quid was considered regular for 77 students (35.56%), sporadic for 45 students (21.11%), and there were 93 students (43.33%) who never chewed it. Regarding eating habits, 138 pupils (64.44%) had a diet heavy in processed foods, whereas 77 students (35.56%) had a diet high in fruits and vegetables.

**Table 2:** Lifestyle factors related to oral cancer risk

Risk factor		Azra Naheed Dental College (n = 215)	Rahbar College of Dentistry (n = 215)
Tobacco use	Using currently	69 (32.22%)	62 (28.89%)
	Former use	31 (14.44%)	19 (8.89%)
	Never used	115 (53.33%)	134 (62.22%)
Betel quid chewing	Regular chewing	93 (43.33%)	77 (35.56%)
	Occasional chewing	43 (20.00%)	45 (21.11%)
	Never chewed	79 (36.67%)	93 (43.33%)
Dietary habits	High in fruits and vegetables	67 (31.11%)	77 (35.56%)
	High in processed foods	148 (68.89%)	138 (64.44%)

Out of the 215 students at Azra Naheed Dental College, 143 (66.51%) had a high awareness of oral cancer, 48 (22.33%) had moderate knowledge, and 24 (11.16%) had poor awareness (Table 3); 167 students (77.67%) were aware of danger factors, while 48 students (22.33%) were not. Comparatively, 131 students (60.93%) at Rahbar College of Dentistry (n = 215) had a high degree of awareness, 60 students (27.91%) had moderate awareness levels, and 24 students (11.16%) had a low level of awareness. A total of 156 students (72.56%) possessed knowledge of risk factors, whereas 59 students (27.44%) did not possess this information.

**Table 3:** Awareness levels of oral cancer among students

Awareness aspect		Azra Naheed Dental College (n = 215)	Rahbar College of Dentistry (n = 215)
Awareness level	High	143 (66.51%)	131 (60.93%)
	Moderate	48 (22.33%)	60 (27.91%)
	Low	24 (11.16%)	24 (11.16%)
Knowledge of risk factors	Yes	167 (7767%)	156 (72.56%)
	No	48 (22.33%)	59 (27.44%)

**Figure 1:**
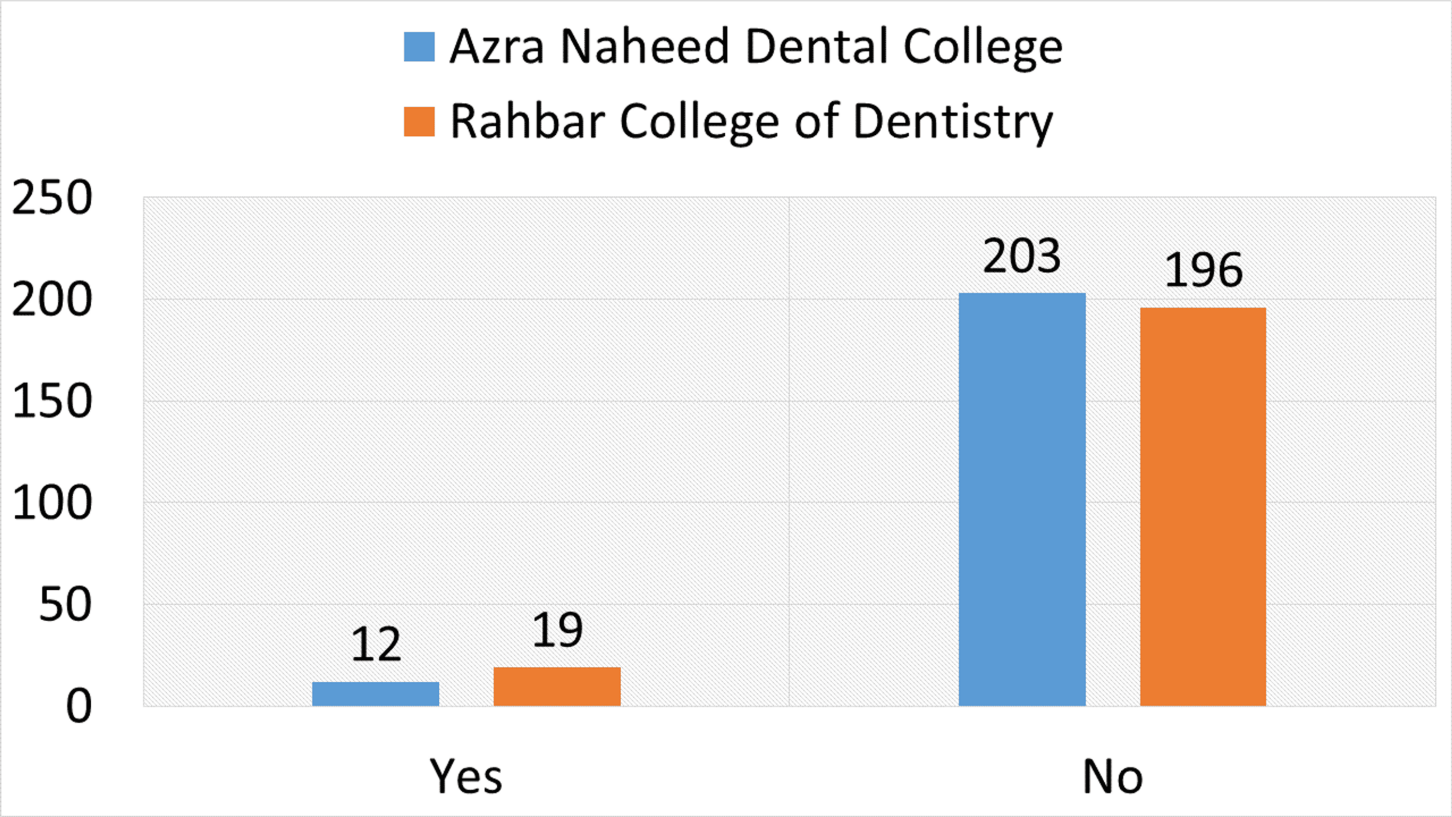
Clinical examination findings for oral cancer The number of students with and without oral lesions at Azra Naheed Dental College and Rahbar College of Dentistry

The results of the clinical examination for oral cancer in students are shown in Figure 1. Twelve students (5.58%) at Azra Naheed Dental College (n = 215) had oral lesions, while 93 students (94.42%) did not. Of the 215 students at Rahbar College of Dentistry, 19 (8.84%) had oral lesions, whereas 196 (91.16%) did not.

The findings of the multivariate logistic regression analysis assessing risk factors for oral cancer are shown in Table 4. There was a strong correlation between oral cancer risk and current tobacco use, with an odds ratio (OR) of 2.15 (95% CI: 1.35 - 3.40, p = 0.001). There was no significant correlation between current tobacco use and outcomes (OR = 1.40, 95% CI: 0.76 - 2.56, p = 0.281). Chewing betel quid regularly was significantly linked to an increased risk of oral cancer (OR = 2.87, 95% CI: 1.76 - 4.71, p < 0.001), although chewing on occasion had no discernible impact (OR = 1.23, 95% CI: 0.78 - 1.92, p = 0.373). While a diet heavy in fruits and vegetables had no discernible protective impact (OR = 0.85, 95% CI: 0.52 - 1.40, p = 0.527), a diet high in processed foods was linked to an elevated risk (OR = 1.62, 95% CI: 1.07 - 2.45, p = 0.021).

**Table 4:** Multivariate logistic regression analysis of risk factors and oral cancer prevalence

Risk factor		Odds ratio (OR)	95% Confidence interval (CI)	p-value
Tobacco use	Currently use	2.15	1.35 - 3.40	0.001
	Former use	1.40	0.76 - 2.56	0.281
Betel quid chewing	Regular chewing	2.87	1.76 - 4.71	<0.001
	Occasional chewing	1.23	0.78 - 1.92	0.373
Dietary habits	High in processed foods	1.62	1.07 - 2.45	0.021
	High in fruits and vegetables	0.85	0.52 - 1.40	0.527

## Discussion

The findings of this study highlight significant risk factors for oral cancer among dentistry college students in Pakistan. The prevalence of oral lesions observed in our study may seem unusually high for this adolescent population; however, it is essential to clarify that these figures reflect the detection of oral lesions, which encompass a range of conditions, not all of which are malignant. Confirmatory biopsies would be necessary to establish a definitive diagnosis of oral cancer, and we acknowledge that the clinical diagnosis can only suggest the presence of malignancy.

Given the established risk factors, such as tobacco use, betel quid chewing, and a diet high in processed foods, it is crucial to develop targeted preventive and educational initiatives to mitigate these risks. Our findings call for further research to validate these prevalence rates and explore the underlying factors contributing to the observed rates of oral lesions.

This cross-sectional study was conducted at two locations, Rahbar College of Dentistry and Azra Naheed Dental College, over six months, involving 430 students aged between 18 and 30 years. This diverse sample allowed for a more comprehensive analysis of the incidence of oral cancer and related risk factors among Pakistani students. The inclusion criteria ensured that we focused on students currently enrolled and excluded those with a history of oral cancer or serious oral disorders, providing a clearer picture of the prevalent risk factors in this demographic.

The variance in prevalence is consistent with other research showing geographical differences in Pakistan's incidence of oral cancer. Similar prevalence rates were discovered in different locations by Anwar et al. [12], indicating that regional variables may affect the incidence of oral cancer. The increased frequency seen at Rahbar College may be related to regional variations in lifestyle characteristics or socioeconomic backgrounds.

The two colleges were found to vary significantly in terms of lifestyle characteristics. While Rahbar College had 28.89% of students who were current smokers, Azra Naheed Dental College had 32.22% of students who were smokers. Tobacco smoking is a well-established risk factor for oral cancer and plays a crucial role in the disease's development [13, 14]. Additionally, 43.33% of students at Azra Naheed Dental College and 35.56% at Rahbar College reported regularly chewing betel quid, with greater prevalence shown to be associated with an elevated risk of oral cancer, as supported by earlier research [15]. Furthermore, 68.89% of students at Azra Naheed Dental College and 64.44% at Rahbar College had diets rich in processed foods, indicating a substantial relationship with oral cancer risk as reported by previous research [16]. The standardized questionnaire utilized in our study effectively quantified these dietary habits and lifestyle factors, providing a comprehensive insight into their patterns related to oral health and cancer risk.

At Azra Naheed Dental College, 66.51% of students reported having a good degree of awareness of oral cancer, compared to 60.93% at Rahbar College. Additionally, 77.67% of students at Azra Naheed were aware of risk factors, compared to 72.56% at Rahbar College. This discrepancy in awareness aligns with other studies that discovered a positive correlation between improved awareness, preventative actions, and greater educational attainment and health education [17]. Despite higher awareness at Azra Naheed, a significant percentage of students at both institutions lacked thorough understanding, suggesting the need for improved educational initiatives.

Multivariate logistic regression analysis showed that current tobacco usage (OR 2.15, 95% CI: 1.35- 3.40, p = 0.001) and frequent betel quid chewing (OR 2.87, 95% CI: 1.76- 4.71, p < 0.001) were significantly linked with a higher risk of oral cancer. These results demonstrate the substantial influence of these risk variables on the incidence of oral cancer and are in line with other research investigations [18, 19]. Moreover, diets high in processed foods also significantly increased the risk of oral cancer (OR 1.62, 95% CI: 1.07 - 2.45, p = 0.021), consistent with previous studies showing a link between diet and cancer risk [20]. This research emphasizes the importance of focused interventions targeting specific risk factors and raising oral cancer awareness in various educational contexts. Subsequent investigations should delve into the fundamental reasons for regional variations and assess the efficacy of proactive measures.

Limitations

The cross-sectional design of this study makes it difficult to establish causal relationships between risk factors and the incidence of oral cancer. Furthermore, reliance on self-reported data for lifestyle characteristics, such as tobacco use and betel quid chewing, may introduce memory bias or inaccuracies. The study's focus on two private dentistry institutions in Lahore may not accurately represent the broader student population across Pakistan. Additionally, while the sample size of 430 students (215 from each institution) offers a reasonable basis for analysis, it may still impact the generalizability of the results. To address these limitations and provide a more comprehensive understanding of oral cancer risk factors, future research should consider longitudinal studies, larger and more diverse populations, and the use of objective measures, such as histological confirmation of diagnoses.

Strengths

This study provides a detailed analysis of specific risk factors associated with oral cancer among dentistry college students, highlighting the significance of lifestyle choices such as tobacco use and betel quid chewing, which can inform targeted interventions and educational initiatives. Utilizing a standardized questionnaire and conducting clinical examinations by trained professionals enhances the reliability and validity of the data collected, ensuring a systematic approach to assessing both dietary habits and the presence of oral lesions in the participant population. The inclusion of a diverse student body from two institutions further strengthens the analysis by allowing for comparisons in the incidence of oral cancer and related risk factors among different demographics and lifestyles.

## Conclusions

This research highlights differences in lifestyle behaviors and awareness among students at private dentistry schools in Lahore, revealing a significant prevalence of risk factors for oral cancer within these institutions. The results demonstrate a clear correlation between frequent chewing of betel quid and current tobacco usage with an elevated risk of oral cancer, specifically SCC. Despite higher levels of awareness at one institution, gaps still exist in understanding among students regarding the risks associated with oral cancer. This underscores the urgent need for improved educational initiatives and targeted preventive measures. In similar educational settings, addressing these gaps through comprehensive health education and tailored interventions should contribute to reducing risk factors and improving oral cancer outcomes.
